# Bioinformatics Analysis Identify Novel OB Fold Protein Coding Genes in *C. elegans*

**DOI:** 10.1371/journal.pone.0062204

**Published:** 2013-04-25

**Authors:** Daryanaz Dargahi, David Baillie, Frederic Pio

**Affiliations:** Molecular Biology and Biochemistry Department, Simon Fraser University, Burnaby, British Columbia, Canada; Inserm U869, France

## Abstract

**Background:**

The *C. elegans* genome has been extensively annotated by the WormBase consortium that uses state of the art bioinformatics pipelines, functional genomics and manual curation approaches. As a result, the identification of novel genes *in silico* in this model organism is becoming more challenging requiring new approaches. The Oligonucleotide-oligosaccharide binding (OB) fold is a highly divergent protein family, in which protein sequences, in spite of having the same fold, share very little sequence identity (5–25%). Therefore, evidence from sequence-based annotation may not be sufficient to identify all the members of this family. In *C. elegans*, the number of OB-fold proteins reported is remarkably low (n = 46) compared to other evolutionary-related eukaryotes, such as yeast *S. cerevisiae* (n = 344) or fruit fly *D. melanogaster* (n = 84). Gene loss during evolution or differences in the level of annotation for this protein family, may explain these discrepancies.

**Methodology/Principal Findings:**

This study examines the possibility that novel OB-fold coding genes exist in the worm. We developed a bioinformatics approach that uses the most sensitive sequence-sequence, sequence-profile and profile-profile similarity search methods followed by 3D-structure prediction as a filtering step to eliminate false positive candidate sequences. We have predicted 18 coding genes containing the OB-fold that have remarkably partially been characterized in *C. elegans*.

**Conclusions/Significance:**

This study raises the possibility that the annotation of highly divergent protein fold families can be improved in *C. elegans*. Similar strategies could be implemented for large scale analysis by the WormBase consortium when novel versions of the genome sequence of *C. elegans*, or other evolutionary related species are being released. This approach is of general interest to the scientific community since it can be used to annotate any genome.

## Introduction

Bioinformatics analysis of the complete genome sequence of *C. elegans* by the WormBase consortium initially revealed over 19000 coding genes [1]. When the genome of the closely related species *C. briggsae* was sequenced and a comparative analysis was performed between the two species, 6% more coding genes were predicted (20261) [2]. Since the bioinformatics annotation pipeline from the WormBase consortium is constantly evolving new protein-coding genes are being predicted and this number is increasing. The latest version of the *C. elegans* genome sequence (WS228) predicts 24610 coding genes. [3] Considering that twice the number of new genes has been predicted using gene prediction algorithms, novel approaches that explore different search spaces may reveal even more protein-coding genes.

Indeed, evidence suggests that more protein may exist in *C. elegans* in the case of old protein fold families that evolved a long time ago from divergent (or convergent) evolution [4]. Such protein family members are renowned to be difficult to identify by conventional sequence alignment software since they share very little sequence identity. The OB-fold is one example [5]. The domain is a compact structural motif frequently used for nucleic acid recognition. It is composed of a five-stranded beta-sheet forming a closed beta-barrel. This barrel is capped by an alpha-helix located between the third and fourth strands. Structural comparison and analysis of all OB-fold/nucleic acid complexes solved to date confirms the low degree of sequence similarity among members of this family arising from divergent evolution [6]. In addition, loops connecting the secondary-structure elements are highly variable in length making them difficult to compare at the sequence level. In *C. elegans* the number of predicted proteins containing OB-fold is remarkably low compared to other related organisms by evolution. The number of OB-fold proteins when we started this project, varied widely from 256 (human), 246 (mouse), 344 (yeast - *Saccharomyces cerevisiae*) to 84 (fruit fly - *Drosophila melanogaster*) and 46 (*C. elegans*). Gene loss or expansion between these different related organisms may have occurred or differences in the level of annotation for this protein family may explain these numerical discrepancies.

The identification of distant related sequences or remote homologues from functional domain families has been extensively improved this past decade. Sequence-sequence and sequence-profile alignment algorithms [7], [8], BLAST [9] and PSI-BLAST [10] have been widely adopted for this purpose. Methods that can detect intermediate sequence to connect sequences sharing insignificant BLAST scores between each other have been implemented [7], [8]. The sensitivity and alignment quality depend on the information that is used to compare proteins. The most sensitive methods use sequence-profiles or profile-profile alignments (Table 1, Sequence Discovery Module). They contain position-specific substitution scores that are computed from the frequencies of amino acids at each position of a multiple alignment of related sequences. Further improvements have been feasible by the introduction of Hidden Markov Models [11], [12] that can compute more accurately gap, insertion and deletion in the alignments compared to previous methods. Moreover, fold recognition methods that build a 3D-structural model of a protein sequence from a sequence alignment have been very efficient in their ability to align correctly sequence/profile to profile of known structures (Table 1, Structure Discovery Module). Building models that are very similar structurally to the templates structure from these alignments can be used to validate a correct alignment, especially if such alignment is between sequences that have very low sequence similarities. More recently, many bioinformatics studies suggest that consensus methods that pool together the results of different software that perform similar tasks perform better than isolated methods.

**Table 1:** Tools used in this study.

Tools	Description	Reference
**Sequence Discovery Module**		
PSI-BLAST	Position-Specific Iterative Basic Local Alignment Search Tool	*Altschul et al. 1990* [9]
MEME	Motif based Hidden Markov Model of protein families	*Grundy et al. 1997* [15]
HMMER	Bio-sequence analysis tool using profile hidden Markov Models	*Eddy, 1996, 1998* [11], [12]
HHpred	Homology detection & structure prediction tool by HMM-HMM comparison	*Soding et al. 2005* [29]
COMPASS	Alignment tool of multiple protein sequence profiles	*Sadreyev et al. 2007* [30]
HHsenser	Exhaustive intermediate profile search tool using HMM-HMM comparison	*Soding et al. 2006* [23]
Saturated-BLAST	Automated toolbox that implement the multiple intermediate sequence search method	*Li et al. 2000* [7]
**Structure Discovery Module**		
MetaServer	A Server that submit and collect fold recognition results from different methods andmakes 3D-prediction using a consensus approach called 3D-jury.	*Bujnicki et al. 2001* [24]
I-Tasser	Protein 3D-structure prediction server that uses threading methods	*Roy et al. 2010* [27]
Modeller	Protein 3D-structure modeling tool from target-template sequence alignment based on satisfaction of spatial restraints	*Fiser et al. 2003* [26]
TM-Align	Protein 3D-structure alignment algorithm that compute the TM-Score	*Zhang et al. 2005* [28]
**Functional Discovery Module**		
BioGrid	Database of Protein and Genetic Interactions	*Stark et al. 2006* [22]
STRING	Database of Functional protein association networks	*Snel et al. 2000* [21]
Worm Interactome	A high quality yeast two-hybrid protein-protein interactions database of *C. elegans*	*Li et al. 2004* [31]
WoLF PSORT	Protein sub-cellular localization predictor	*Horton et al. 2007* [32]
Kihara PFP	Protein function predictor	*Hawkins et al. 2006* [33]

This study examines the possibility that novel OB-fold coding genes exist in the worm. We developed a consensus approach that uses the most sensitive sequence-sequence, sequence-profile and profile-profile similarity search methods followed by OB-fold 3D-structure prediction as a filter to eliminate false positive candidate remote sequences. We have predicted 18 coding gene containing the OB-fold. Remarkably, most of their corresponding genes have not been or have only been partially characterized in the worm. As expected, many of them are essential genes since their knockout produces lethal phenotypes. And it is well known that OB-fold containing proteins are frequently involved in essential nucleic-acids metabolism, such as Replication Protein A [13], tRNA synthetases [14].

## Results

Using the profiles generated by MEME [15] and PSI-BLAST [10] from the 46 proteins sequences annotated as OB-fold in the *C. elegans* genome we obtained an additional 200 candidate proteins that may contain OB-fold (see methods). We attempted to validate these with structural alignment programs such as MetaServer, I-Tasser, Modeller and TM-align, but only two (brc-2 and pot-1) were predicted to be good structural maps to the OB-fold by any of these methods. This finding was not far from our expectation since many OB-fold family members share less than 10% sequence similarity between each other, which is consistent with the high degree of sequence divergence of this family that occurred during evolution. Therefore, even though very sensitive sequence alignment methods are used, detection of novel OB-fold proteins remained difficult.

Since very divergent sequences that do not share significant sequence identity may have the same fold, and considering the conserved structure of OB-fold, we used fold recognition methods of StrucDiM to investigate if more OB-fold proteins could be obtained directly. The underlying assumption was that if a correct model can be built by comparative modeling using a sequence alignment between a protein sequence of an OB-fold of known structure with an OB-fold candidate sequence, then the sequence alignment is significant. It allows us to put some confidence in the pairwise alignment of sequences that share a level of sequence identity below the twilight zone (18–25% identity) [16], [17], [18] since sequence alignment statistics cannot determine their significance at this level of identity. Effectively, incorrect alignments do not generate well-folded homology models. Since the *C. elegans* genome encodes greater than 20000 genes and many of these genes products would not be of interest, we decided to use a dataset likely to be enriched in genes containing OB-fold 3D-structure. For this purpose, we selected the 4300 genes identified by Claycomb et al. [19] that are expressed in the germline of *C. elegans*. We expected this dataset to be enriched in genes involved in DNA processes, including DNA repair and replication, which may contain protein coding genes with OB-fold 3D-structure and also exclude gene involved in terminal differentiation of tissues such as muscle, nerve, gut or organ that may not be relevant to this study. Each sequence was submitted directly to 3D-structure prediction using StrucDiM.

**Figure 1:**
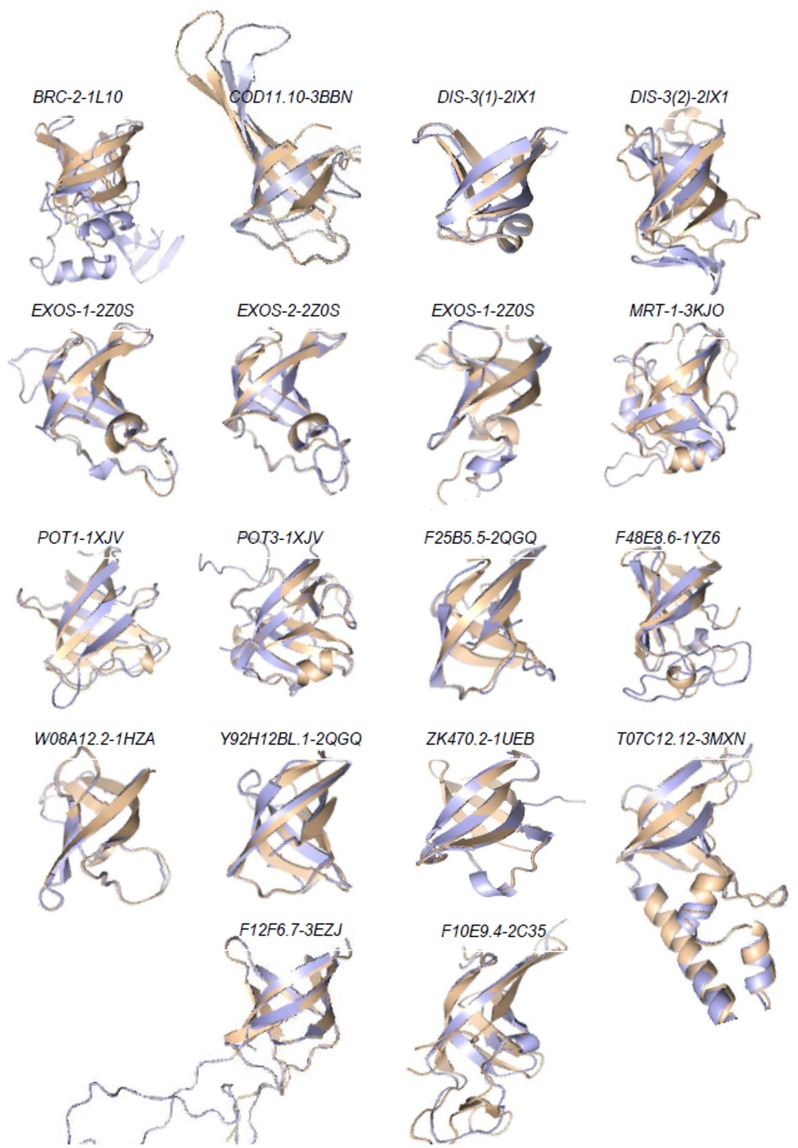
Superimposition of the novel OB-fold 3D-model with their templates. (Light blue): Predicted 3D-models, (Wheat) PDB template. (.XXXX.)-nxxx name correspond to the protein name followed by the PDB code of the template.

By this direct approach, we determined that 35 out of 46 previously annotated OB-fold proteins in the entire genome of *C. elegans* were present in the 4300 germline expressed genes set [19]. Thus, the dataset is clearly enriched in OB-fold sequences (about three fold). It also showed that the StrucDiM approach was valid and could be used to further identify novel OB-fold protein coding genes (Figure 1). Indeed, in addition to the 46 already annotated and known OB-fold proteins we identified 14 novel OB-fold candidate proteins OB-fold (Table 2). However, it should be noted that one of the member of this list, the OB-fold 3D-structure of the human homologue pot-1, has been recently deposited in the Protein Data Bank (PDB accession number: 1XJV). These results show that our approach is highly sensitive to predict novel OB-fold protein candidates. Further, structural and functional studies are needed to assess the specificity of these OB-fold prediction results.

**Table 2:** Model quality of novel OB-fold protein coding genes.

OB-fold Candidates target	Template	RMSD	TM-score	Equivalent C_alpha_ superimposed
**F12F6.7**	3E0J	0.9	0.91618	104/110
**F25B5.5**	2QGQ	1.08	0.79684	57/64
**exos-2**	2Z0S	0.39	0.91855	80/86
**exos-3**	2Z0S	1.33	0.93357	66/66
**exos-1**	2Z0S	0.97	0.83856	76/85
**dis-3 (First OB-fold)**	2IX1	2.15	0.77503	81/92
**dis-3 (Second OB-fold)**	1UEB	3.66	0.51393	76/98
**ZK470.2**	3BBN	1.22	0.90075	43/45
**C05D11.10**	1HZA	1.88	0.8186	77/82
**W08A12.2**	2C35	1.27	0.91183	58/59
**F10E9.4**	1XJV	1.98	0.81487	61/61
**Pot-1**	1L1O	1.11	0.89915	128/135
**brc-2**	1XJV	3.43	0.43998	74/115
**Pot-3**	3MXN	1	0.83903	115/133
**T07C12.12**	1XJV	1.49	0.90455	132/139
**Pot-2**	3KJO	0.4	0.86529	110/126
**mrt-1**	2QGQ	1.03	0.83313	115/135
**Y92H12BL.2**	1YZ6	0.62	0.89597	56/60
**F48E8.6**	3E0J	2.27	0.64349	66/81

To further identify additional OB-fold gene coding proteins we searched for orthologues and homologues of the identified candidates in both human and *C. elegans*. Using the protein family orthologues, and paralogues module in the comparative genomics toolbox of ENSEMBL database we were able to identify 3 additional candidate homologues of pot-3 (pot-2, mrt-1, F48E8.6) and one homologues of F25B5.5 (Y92H12BL.2). We expected to see that these proteins also have OB-fold similar to their paralogues. In addition, we then used structDiM to verify the predicted OB-fold structure of these proteins. As expected, all candidates were confirmed to contain OB-fold. These 4 novel OB-fold proteins had not been previously predicted and annotated in the WormBase, however, for 2 of them (mrt-1 and pot-2) we found one publication mentioning that these two genes contained an OB-fold domain [20].

## Discussion

One important question regarding this study is why the annotation of these genes had been missed from the WormBase database (www.WormBase.org). The obvious lack of sequence similarity among members of this family is one possible explanation since it makes these proteins undetectable through sequence based searches. This is consistent with our inability to identify novel OB-fold protein coding genes using the SeqDiM module. On the contrary, we have showed that structural based methods are more robust at predicting OB-fold proteins. Since these methods are generally not considered in genome annotation pipelines, this may explain why many of these OB-fold containing genes have not been annotated.

Regarding the genes that have been identified, it is remarkable that most of them have not been well studied (Table 3). However, a significant fraction of their gene products perform important function during development and are essential genes since RNAi phenotype (EXOS-3) as well as knockout when available shows embryonic lethality. Those with embryonic lethality include protein coding genes involved in DNA replication and repair (F12F6.7, BRC-2) and growth rate and reproduction (EXOS-1, C05D11.10, F10E9.4) as well as the protection of telomere protein POT-3 involved in telomere maintenance. Other OB-fold candidate proteins do not seem to be essential during development since they only show no or non-lethal phenotypes. Those include gene coding proteins involved in nucleic acids and RNA binding (EXOS-2) a component of the exosome complex (with EXOS-1 and EXOS-3), DIS-3, ZK470.2, W08A12.2, T07C12.12, F25B5.5 as well as POT-1 involved in telomere maintenance. To annotate further the function of these genes, we looked at protein-protein interaction in the STRING [21] and BIOGRID [22] databases. No interactions were found for most of them with the exception of EXOS-3, C05D11.10, POT-1, and BRC-2. These interact respectively with genes products involved in cell division, nucleic-acid binding/RNA processing, IGF signaling/life span extension/longevity for POT-1 and DNA repair for BRC-2.

**Table 3:** Functional analysis of Novel OB folds protein coding genes.

OB folds	WB ID	Biblio	RNAi Phenotype	Knockout	Function	Homologues Paralogues	Paralogs
F12F6.7	WBGene00008722	NA	Embryonic lethal	ok2252	DNA replication, DNA binding,DNA-directed DNA polymerase activity	POLD2 polymerase (DNA directed),delta 2, regulatory subunit 50kDa	NA
F25B5.5	WBGene00017776	NA	NA	NA	RNA modification, iron-sulfur cluster binding,4 iron, 4 sulfur cluster binding, catalytic activity	CDK5RAP1 CDK5 regulatory subunitassociated protein 1	Y92H12BL.2
exos-2	WBGene00022232	[34]	late larval arrest	NA	*nucleic acid binding, RNA binding,	EXOSC2 exosome component 2	NA
exos-3	WBGene00010325	[34]–[36]	Embryonic lethal	NA	growth,nematode larval development, receptor-mediated endocytosis	EXOSC3 exosome component 3	NA
exos-1	WBGene00012966	[34]	Embryonic lethal, lethal	ok807	positive regulation of growth rate,reproduction	EXOSC1 exosome component 1	NA
dis-3	WBGene00001001	[37]–[39]	Slow growth, sick, sterile progeny	ok357	RNA binding, ribonuclease activity, sequence-specific DNA binding, reproduction	DIS3 mitotic control homolog(S. cerevisiae)	F48E8.6
ZK470.2	WBGene00022745	[40]	NA	ok5876	*single-stranded telomeric DNA binding, ion binding, monosaccharide metabolism	NA	NA
C05D11.10	WBGene00015487	NA	Embryonic lethal, lethal, slow growth	ok5298	growth, nematode larval development,positive regulation of growth rate, reproduction	NA	NA
W08A12.2	WBGene00021079	NA	NA	NA	*purine nucleotide binding, adenyl nucleotide binding, cellular macromolecule metabolism	NA	NA
F10E9.4	WBGene00017356	NA	Slow growth, larval lethal	NA	growth, nematode larval development, positive regulation of growth rate, reproduction	NA	NA
Pot-1	WBGene00015105	[20], [41]–[42]	organism development variant, telomere homeostasis variant	NA	*cAMP-dependent protein kinase activity, transition metal ion binding, ion binding	Pot1 Protection Of Telomeres 1	NA
brc-2	WBGene00020316	[43]–[55]	Embryonic lethal, lethal,embryonic arrest	ok1629	strand invasion, double-strand break repair, reproduction, single-stranded DNA and protein binding	Brca1 Breast Cancer type 1 susceptibility protein	NA
Pot-3	WBGene00007065	[22], [44]	lethal	ok1530	*cation binding, adenyl nucleotide binding, heterocycle metabolism	Pot1 Protection Of Telomeres 1	pot-2, mrt-1
T07C12.12	WBGene00011576	[56]	Embryonic lethal	NA	*adenyl nucleotide binding,rRNA (adenine) methyltransferase activity, purine nucleotide binding	RMI1, RecQ mediated genomeinstability 1	NA
Pot-2	WBGene00010195	[20], [42]	NA	NA	*cation binding, adenyl nucleotide binding, heterocycle metabolism	NA	pot-3, mrt-1
MRT-1	WBGene00045237	[20,42,56–59]	Sterile, lethal	oK758	Nuclear excision repair, telomere maintenancevia telomerase, reproduction, Singlestranded DNA binding	NA	pot-2, pot-3
Y92H12BL.2	WBGene00022363	NA	NA	NA	Iron-sulfur cluter binding	CDKAL1, CDK5 regulatory subunitassociated protein 1-like 1	F25B5.5
F48E8.6	WBGene00018612	NA	NA	NA	RNA binding, ribonuclease activity	DIS3L2, DIS3 mitotic control homolog(S. cerevisiae)-like 2	dis-3

* refers to predicted functions. Homologues and paralogues referred to human.

We have shown that comparative modelling approaches are powerful tools to identify novel protein coding genes with interesting and uncharacterized functions even in a genome and proteome of a model organism as extensively annotated as *C. elegans*. Such approach is of general interest to the scientific community since it can be applied to any genome.

## Materials and Methods

### Input Sequences

Protein sequences used in this study to identify novel OB-fold proteins were obtained from the 46 OB-fold known proteins in WormBase and an enriched data set of 4300 expressed genes in the germ line of *C.elegans* [19]. This dataset should be enriched in novel genes containing OB-fold since OB-fold proteins are generally involved in many DNA transaction and repair processes highly actives in the *C. elegans* germline.

### Consensus Discovery Pipeline

**Figure 2:**
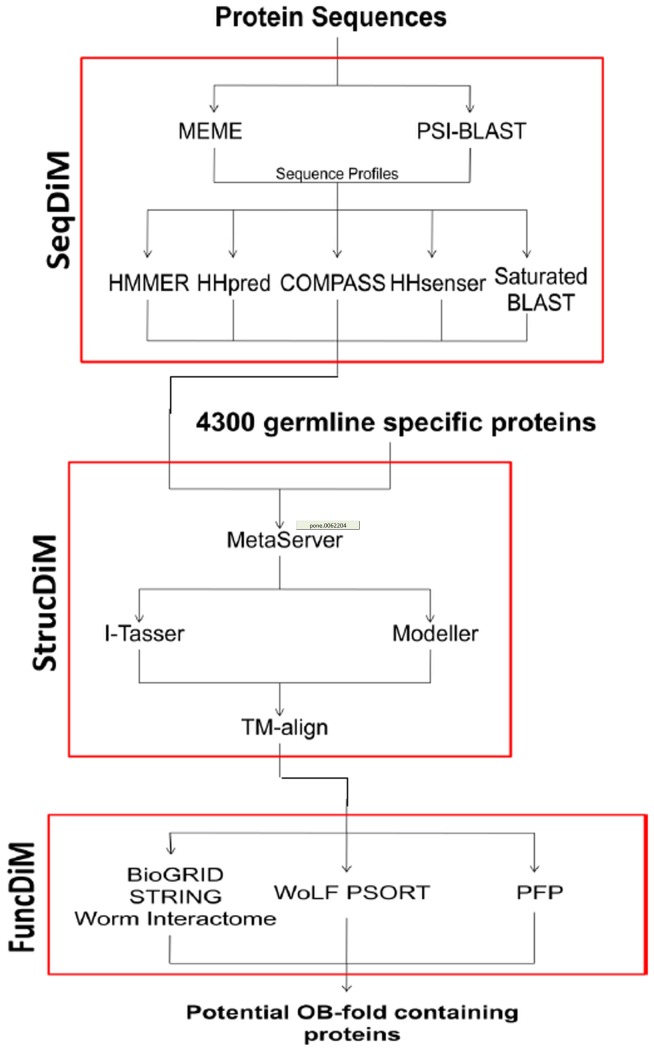
Discovery Pipeline of novel OB fold protein coding genes. It contains 3 Discovery Modules. SeqDIM: Sequence alignment DIscovery Module; StrucDIM:3D Structure prediction Discovery Module; and a Functional prediction Discovery Module FuncDIM.

The pipeline (Figure 2, Table 1) has 3 modules (i) **Seq**uence based **Di**scovery **M**odule (ii) **Struc**ture based **Di**scovery **M**odule and filtering (iii) **Func**tional **Di**scovery **M**odule:

#### Sequence based Discovery Module

From the 46 OB-fold known proteins sequences in *C. elegans* a position-specific scoring matrix of OB-fold motifs was built using PSI-BLAST [10] as well as a Hidden Markov Model using MEME [11], [12], [15] Each of the profiles were subsequently submitted to different database scanning software using sequence-profile based alignment methods against the wormpep210 protein sequence database. For the HHSenser profile-profile methods [23] the database was made-up of sequence profiles of all the known protein families. For each method the default threshold of significance were used to select for novel candidate OB-fold protein sequences (see Text S1, Figure S1 and S2, Table S1 and S2).

#### Structural Discovery Module

The 4300 sequences from claycomb et al. [19] as well as the 200 sequence OB-fold candidates obtained from SeqDiM were submitted to the consensus fold recognition metaserver [24] to perform and confirm fold prediction. This method collects and scores many different fold prediction results using the 3D jury consensus method from a protein sequence [25]. Model building for the predicted OB-fold motif in candidate genes were further performed by the modeller algorithm [26] from meta-server sequence alignment results as well as re-submitting candidate sequences to the 3D-structure prediction server I-tasser [27]. Model quality and validation were further performed using TM-align [28]. A TM-score <0.2 indicated that there was no similarity between two structures; a TM-score >0.5 meant that the structures shared the same fold (Text S1, Figure S3).

#### Functional Discovery Module

To gain insight into the function of the novel OB-fold candidates discovered, protein-protein interaction databases, subcellular localization and gene ontology predictors were interrogated (Table 1, Function Discovery Module).

## Supporting Information

Figure S1 **Generation of PSI-BLAST profiles using the 46** ***C. elegans*** **OB fold protein sequences.**
(TIF)

Figure S2 **Profile based search to identify novel OB fold protein sequences.**
(TIF)

Figure S3 **Direct fold recognition prediction to identify novel OB fold protein.**
(TIF)

Table S1 **Parameters explored for profile generation using PSI-BLAST**
(DOCX)

Table S2 **Parameters of sequence similarity search tools used on step 4. Mostly default parameters were used otherwise specified.**
(JPG)

Text S1 **Supporting methods.**
(DOC)
