# Supplementary figures and images for: Bioinformatics Analysis Identify Novel OB Fold Protein Coding Genes in C. elegans
Source: PLoS One. 2013 Apr 25;8(4):e62204. doi: 10.1371/journal.pone.0062204 (PMC3636199; doi:10.1371/journal.pone.0062204)

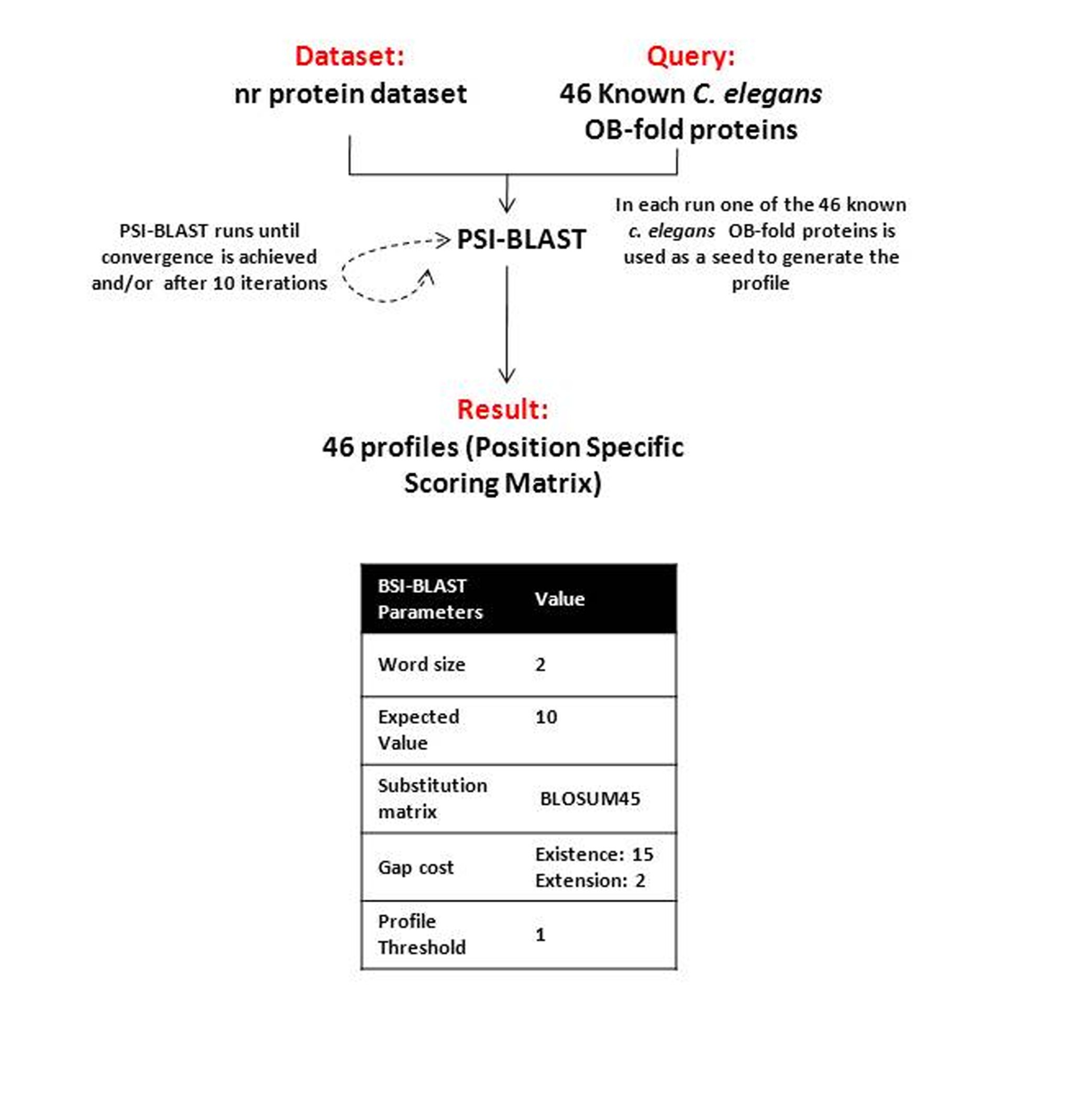

Supplement: Figure S1 — Generation of PSI-BLAST profiles using the 46 C. elegans OB fold protein sequences. (TIF) [file pone.0062204.s001.tif]

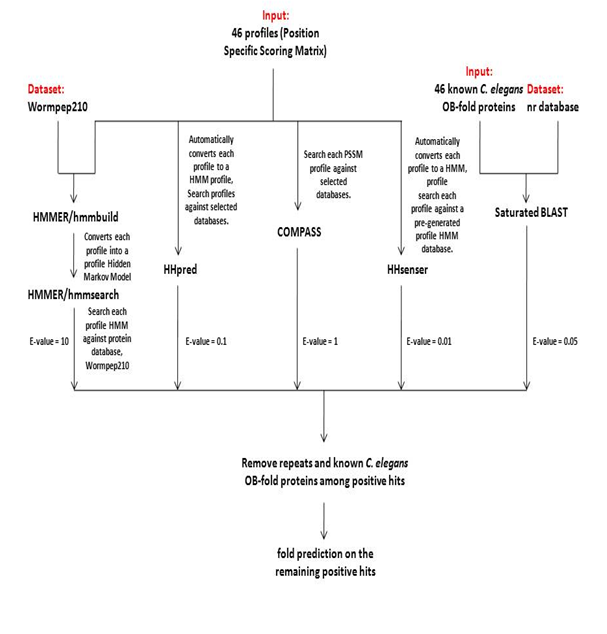

Supplement: Figure S2 — Profile based search to identify novel OB fold protein sequences. (TIF) [file pone.0062204.s002.tif]

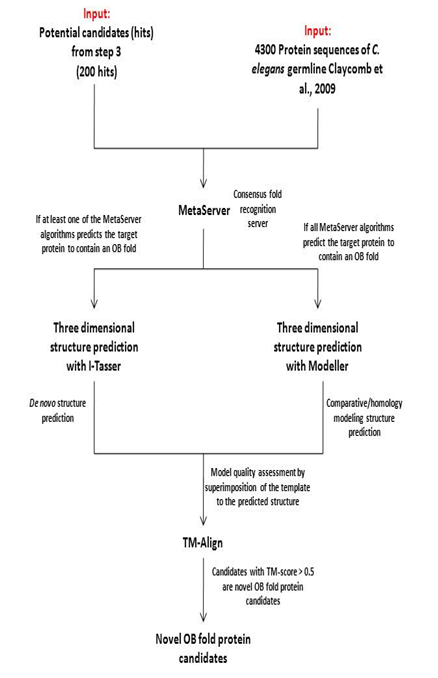

Supplement: Figure S3 — Direct fold recognition prediction to identify novel OB fold protein. (TIF) [file pone.0062204.s003.tif]

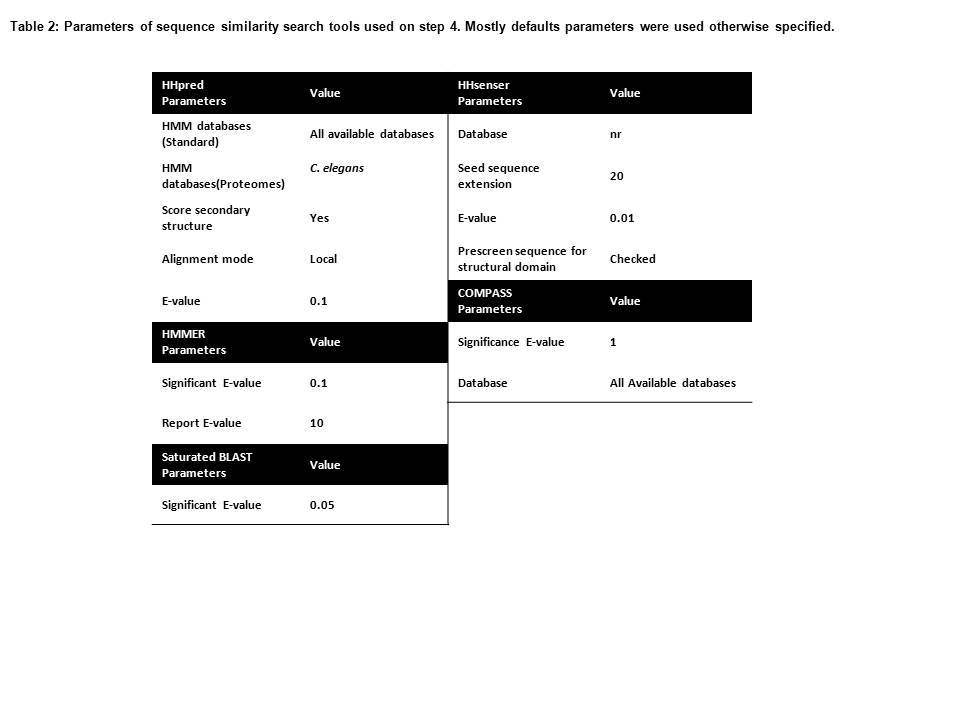

Supplement: Table S2 — Parameters of sequence similarity search tools used on step 4. Mostly default parameters were used otherwise specified. (JPG) [file pone.0062204.s005.jpg]
