# Supplementary material for: Bioinformatics Analysis Identify Novel OB Fold Protein Coding Genes in C. elegans
Source: PLoS One. 2013 Apr 25;8(4):e62204. doi: 10.1371/journal.pone.0062204 (PMC3636199; doi:10.1371/journal.pone.0062204)
Supplement: Table S1 — Parameters explored for profile generation using PSI-BLAST (DOCX) [file pone.0062204.s004.docx]

Table 1. Parameters explored for profile generation using PSI-BLAST.

| **Parameter** | **Default value** | **Altered value** | **Explanation** |
| --- | --- | --- | --- |
| **Word size** | 3 | 2 | Larger word size (WS) increases the probability of missing remote evolutionary relationship and weak similarities. Small word size increase the computational time and the probability of false positives occurrence. |
| **Expected value** | 10 | 10 | At this E-value many alignments may occur by chance but most known OB fold proteins were obtained at this threshold. To distinguish real positive hits from false positive, manual analysis of alignments and further validations of the hits by fold prediction algorithm were performed |
| **Substitution matrix** | BLOSUM62 | BLOSUM45  BLOSUM80 | For remote homology search, BLOSUM was used. To identify closely related sequences BLOSUM80 was used. More distant related sequences were identified using BLOSUM45. |
| **Gap cost** | Existance:11  Extension:1 | B45: 15, 2  B80: 10, 1 | Gap cost depends on the choice of the substitution matrix. It illustrates the cost of opening and extending a gap. |
| **Profile threshold** | 0.005 | 0.0005  0.05  1 | The statistical significant threshold at which a sequence can be included in the profile. Smaller value means selection of closely related sequences while larger values let more distantly related sequences to be included into profile. |
