# Supplementary material for: Bioinformatics Analysis Identify Novel OB Fold Protein Coding Genes in C. elegans
Source: PLoS One. 2013 Apr 25;8(4):e62204. doi: 10.1371/journal.pone.0062204 (PMC3636199; doi:10.1371/journal.pone.0062204)
Supplement: Text S1 — Supporting methods. (DOC) [file pone.0062204.s006.doc]

**Supplementary methods**

**Steps in the discovery pipeline**

**[1]** 46 known *C. elegans* OB-fold proteins sequences were obtained from WormBase database.

**[2]** For each OB-fold protein sequences a PSSM matrix were made using PSI-BLAST (46 PSSM matrices in total). PSI-BLAST was run using an expected threshold of 10 and a P-value of 0.5. PSI-BLAST was run until convergence is obtained or stopped at a maximum of 10 iterations. In each round, new hits were obtained using E-value, Max identity, and available GO annotation parameters from Table 1. At this E-value and P-value high level of divergence OB-fold can be identified since these proteins often share very low sequence similarity. A true hit might appear with high P-/E- value (Figure S1).

**[3]** In parallel, since profile methods are highly sensitive and specific to detect divergent sequences we used five well-known algorithms HMMER, HHpred, COMPASS, HHsenser, and Saturated BLAST. Profiles generated from step [2] were used as input to build different types of profiles and scan a database with each of them to search for novel OB-fold proteins (Figure S2, Table S2).

- HMMER was run using the default parameters with an E-value = 0.01, and a report E-value = 1 against the Wormpep210 database downloaded from Wormbase database. The Hidden Markov Model profiles were built from the PSI-BLAST multiple sequence alignments generated in step [2] using the HMMbuild software. Database scanning of Wormpep210 was performed using HMMsearch.
- HHpred was run by the default parameters using the options HMM databases = all available databases, Proteomes = *C. elegans*, secondary structure score = Yes, Alignment mode = Local, and E value = 0.1.
- HHsenser was run by the default parameters using the options database = nr, seed sequence extension = 20, E value = 0.01, and prescreen sequence for structural domain = checked. HHsenser search for OB fold proteins against a pre-compiled profile-HMM database.
- COMPASS was run using the parameters values and options E-value = 1, significance threshold = 0.05, database = all iterations. The algorithm was run by searching with the submitted alignments produced in step [2] as a query against a numerical database of profiles that include SCOP40, PDB70, PFAM and KOG databases.
- Saturated BLAST was run by default parameters including significance threshold = 0.05 against wormpep210.
- Step [3] identified 200 protein sequences potentially related to OB fold family. Most of the known *C. elegans* OB-fold proteins sequences were also present in the search outputs indicating that the threshold value were appropriate to detect novel OB fold protein sequences.

**[4]** False positive OB fold protein sequences from the 200 proteins identified in step 3 were filtered using the MetaServer, a comprehensive fold recognition prediction web resource. Sequences that were not predicted to contain OB fold in their sequence were considered false positive. The MetaServer interrogate several fold prediction algorithms and report a consensus score to rank the best hits according to the 3D-jury method developed by *Ginalski et al. 2003*. If none of the algorithms predict OB-fold for a sequence, the sequence was removed from the list. When only one or few algorithms predicted OB fold, the query sequence was further confirmed using a 3D structure prediction algorithms not being used by the metaserver such as I-tasser.

**[5]** Due to the high sequence divergence of OB fold proteins, it is possible that some of the homologues were not identified through sequence based search. As a result we decided to identify additional OB fold proteins through direct structural search from a dataset that maybe enriched in OB fold containing proteins (figure S3). We used the recently published dataset of proteins expressed in the germline of *C. elegans* by *Claycomb et al. 2009*. 4300 germline specific proteins were submitted to the MetaServer. If at least one of the MetaServer’s fold prediction algorithms predicted OB-fold for the query protein, it was further confirmed by 3D structure prediction using i-tasser. On the negative the sequence were removed from the list.

**[6]** Model building of the positive hits identified by the metaserver were performed using Modeller from the metaserver website as well as re-submitting candidate sequences to the 3D-structure prediction server I-Tasser (default parameters were used for both I-Tasser and Modeller). Model quality was investigated using TM-align. A TM-score < 0.2 indicated that there was no similarity between two structures; while a TM-score > 0.5 meant that the structures shared the same fold. Hence, candidates with TM-Score > 0.5 were considered as novel OB-fold proteins. (Figure S3)

**[7]** To gain insight into the function of the novel OB-folds discovered available Gene Ontology, protein-protein interaction and subcellular localization were investigated. If no information were available we predicted the function and localization of the candidate using the Protein Function Prediction tool (PFP) and WoLF PSORT, respectively. Both tools were run by default parameters.
